# Comparison of Inflammatory and Angiogenic Factors in the Aqueous Humor of Vitrectomized and Non-Vitrectomized Eyes in Diabetic Macular Edema Patients

**DOI:** 10.3389/fmed.2021.699254

**Published:** 2021-09-08

**Authors:** Youling Liang, Bin Yan, Zhishang Meng, Manyun Xie, Zhou Liang, Ziyi Zhu, Yongan Meng, Jiayue Ma, Bosheng Ma, Xiaoxi Yao, Jing Luo

**Affiliations:** ^1^Department of Ophthalmology, The Second Xiangya Hospital, Central South University, Changsha, China; ^2^Shenzhen College of International Education, Shenzhen, China

**Keywords:** central retinal thickness, diabetic macula edema, inflammation, post-operative DME, VEGF

## Abstract

**Objectives:** To compare the aqueous concentrations of inflammatory and angiogenetic factors in vitrectomized vs. non-vitrectomized eyes with diabetic macular edema (DME).

**Methods:** Aqueous samples were obtained from 107 eyes with DME before intravitreal injection of anti-VEGF, 36 eyes with previous pars plana vitrectomy (PPV) combined with pan-retinal endolaser photocoagulation (PRP), and 71 treatment-naïve. Interleukin (IL)-6, IL-8, interferon-induced protein (IP)-10, monocyte chemoattractant protein (MCP)-1, and vascular endothelial growth factor (VEGF) were measured by cytometric bead array (CBA). Optical coherence tomography (OCT) was used for measuring central retinal thickness (CRT).

**Results:** IL-6, IL-8, IP-10, and MCP-1 in aqueous humor of DME vitrectomized eyes were significantly higher than in non-vitrectomized DME eyes, while VEGF was lower than in non-vitrectomized DME eyes. VEGF in aqueous humor significantly correlated with CRT for DME in non-vitrectomized DME eyes. IL-6, IL-8, IP-10, and MCP-1 in aqueous humor were not significantly associated with VEGF for DME in vitrectomized eyes.

**Conclusions:** Inflammation might play an important role in the pathogenesis of DME in vitrectomized eyes. Moreover, inflammation might play a central role in the development of DME *via* the VEGF-independent pathway. Thus, anti-inflammatory therapy might be a strategy for DME in vitrectomized eyes.

## Introduction

Anti-VEGF intravitreous injection is emerging as first-line therapy for diabetic macular edema (DME) (1). Still, the effectiveness of anti-VEGF therapy in eyes after pars plana vitrectomy (PPV) remains uncertain. PPV is a treatment for advanced proliferative diabetic retinopathy (PDR) (2), but many patients still suffer from DME post-PPV, and no consensus has been reached regarding the optimal treatment for those patients.

The study by Yanyali et al. (3) demonstrated that anti-VEGF therapy in vitrectomized eyes with DME had no effect on visual acuity or foveal thickness, which indicated that the mechanism of DME post-PPV might be different from DME without surgery due to the changes in the microenvironment. Inflammation is also an important contributor to DME pathogenesis (4), especially when the blood–retina barrier is broken by the surgery. Thus, inflammation might be an important reason for post-operative DME. Interleukin (IL)-6 is a pro-inflammatory cytokine associated with the blood-ocular barrier; therefore, increased IL-6 might increase endothelial permeability by rearranging actin filaments and changing the shape of the endothelial cells (5), leading to the leakage of fluorescein in the macula (6). In addition, IL-6 levels are associated with the recurrence of DME after anti-VEGF treatment (7). IL-8 is believed to be involved in inflammation-mediated angiogenesis and serves as a fundamental factor in the inflammatory basis of diabetic retinopathy (DR) (8). The vitreous levels of IL-8 are elevated in DME compared with non-DME eyes (7, 9). Interferon-induced protein (IP)-10 is known to inhibit neovascularizations (10). IP-10 can prevent IL-8-mediated angiogenesis and prevent the progression of PDR (11, 12). MCP-1 is a chemotactic chemokine that induces monocyte and macrophage infiltration into tissue (13) and might have a tight relationship with the process of fibroproliferation (14). Aqueous MCP-1 levels are elevated in DME eyes (15).

In order to investigate and analyze the role of inflammatory factors in post-operative DME, we compared the levels of the inflammatory cytokines mentioned above and VEGF in the aqueous humor of vitrectomized vs. non-vitrectomized DME eyes. The purpose of this study was to determine the optimal treatment for post-operative DME patients. Steroids, including slow-release steroids, might be an option for DME in vitrectomized eyes if the inflammation factors are proven to be an important factor in DME after PPV.

## Methods

### Patients

This study was approved by the Ethical Committee of The Second Xiangya Hospital (LYZ2020009), and all enrolled patients were treated in accordance with the Declaration of Helsinki. All patients provided informed consent before inclusion in the study. To reduce selection bias, all patients were recruited consecutively.

We obtained undiluted aqueous humor samples from 109 DR patients (109 eyes) prior to receiving an anti-VEGF intravitreal injection, including 38 eyes that had previously undergone PPV combined with pan-retinal endolaser photocoagulation (PRP) due to vitreous hemorrhage (referred to as the vitrectomized group), and 71 treatment-naïve eyes (referred to as the non-vitrectomized group). The PPV-combined PRP in the vitrectomized group was performed at The Second Xiangya Hospital of Central South University (Hunan province, China) by the same surgeon.

The inclusion criteria were:

- patients aged 18–80 years with type 2 DM
- diagnosed with PDR
- best correct visual acuity <20/40
- DME involving macular
- vitrectomized group: PPV was performed at least 4 weeks before enrollment
- non-vitrectomized group: all patients were treatment-naïve

The exclusion criteria were:

- vitrectomized group: intravitreous injection after PPV
- non-vitrectomized group: prior ocular surgery, including intravitreous injection
- any history of ocular inflammation
- an abnormal vitreoretinal interface
- history of renal and hematologic diseases, uremia, prior chemotherapy, and chronic diseases other than diabetes (renal disease and hematological disease).

### Sampling Procedure

Aqueous humor samples (50–100 μl) from patients with DME were obtained by anterior chamber paracentesis at the beginning of the vitreous injection. The specimens were manually aspirated using a 30-gauge needle and then transferred immediately into sterile tubes. The scheduled vitreous injections were subsequently performed. All samples were stored at −80°C until assayed.

### Testing of Macular Edema

Central retinal thickness (CRT) was measured using spectral-domain optical coherence tomography (OCT; RTVue XR Avanti, Optovue, Inc., Fremont, CA, USA) in all included eyes before the collection of aqueous humor samples. A macular profile of the central 6-mm zone was obtained using the fast macular scan protocol. CRT was calculated by extrapolating radial measurements as an average value within a circle with a 500-μm radius centered on the fovea. In the presence of vitreoretinal traction or epiretinal membrane, an OCT signal strength <4, or the OCT featured retinal border detection algorithm artifacts, the OCT result was excluded from the analysis.

### Cytokine Detection

The quantitative determination of IL-6, IL-8, IP-10, MCP-1, and VEGF levels in the aqueous humor samples was performed using the CBA Human Cytokine kit (BD Biosciences; San Diego, CA, USA), according to the manufacturer's protocol. A sample volume of 10 μl (either the standards or the patient samples) was added to 50 μl of a cocktail consisting of capture beads and detector antibodies, and the mixture was incubated for 18 h at room temperature in the dark. Before data acquisition, the excess unbound detector antibody was washed off. For statistical analyses, a measured value below the threshold of detection was conservatively omitted from the analysis.

### Statistical Analysis

Statistical analysis was performed using SPSS 24.0 (IBM, Armonk, NY, USA). The results are presented as the mean ± SD. The mean cytokine levels, as detected by CBA, were compared between groups using the Mann–Whitney *U*-test. Spearman's correlation analysis was used to examine the correlations between cytokine concentrations and CRT values. *p*-values < 0.01 were considered significant when comparing data between two groups, and Bonferroni-corrected alphas were used for the evaluation of the results. There were two groups, and five pairwise tests were performed. The resulting *p*-values were evaluated at an alpha = 0.01 and with a *p*-value < 0.01 which was judged significant. When doing correlation analysis, we will find a significant *p*-value after Ryan–Holm step-down Bonferroni correction.

## Results

The clinical characteristics of all patients are shown in Table 1. Two samples fell below the detection threshold; therefore, all data associated with these two eyes were omitted from the study. The number of enrolled eyes in the vitrectomized group was 36 (15 men and 21 women), and the number of enrolled eyes in the non-vitrectomized group was 71 (32 men and 39 women). The mean duration of DM in the vitrectomized group was 11.7 ± 6.6 years compared with 9.1 ± 6.9 years in the non-vitrectomized group. In addition, the mean glycosylated hemoglobin (HbA1c) levels were similar in the two groups.

**Table 1:** Clinical and laboratory characteristics of the patients.

	**Vitrectomized group**	**Non-vitrectomized group**	***p*-value**
Age (years)	53.6 ± 6.4	56.7 ± 8.2	0.051
**Sex (** ***n*** **)**			0.740
Male	15	32	
Female	21	39	
Duration of diabetes (years)	11.7 ± 6.6	9.1 ± 6.9	0.059
Glycosylated hemoglobin (%)	7.26 ± 0.89	7.78 ± 1.49	0.056

The mean concentrations of IL-6, IL-8, IP-10, and MCP-1 were significantly higher in the vitrectomized group than those in the non-vitrectomized group (*p* < 0.001). However, the levels of VEGF were significantly lower in the vitrectomized group than in the non-vitrectomized group (*p* = 0.001; Table 2).

**Table 2:** Levels of inflammatory cytokines and VEGF in the aqueous humor of vitrectomized and non-vitrectomized groups.

	**Vitrectomized group**	**Non-vitrectomized group**	***p*-value**
IL-6	288.60 ± 392.28	44.43 ± 83.88	<0.001^*^
IL-8	50.08 ± 34.30	18.27 ± 17.37	<0.001^*^
IP-10	154.99 ± 162.15	50.59 ± 69.17	<0.001^*^
MCP-1	1650.45 ± 911.02	712.07 ± 429.57	<0.001^*^
VEGF	36.18 ± 28.95	72.44 ± 62.67	0.001^*^

* *Statistical significance: p < 0.01 determined by Mann–Whitney U test*.

*IL-6, interleukin-6; IL-8, interleukin-8; IP-10, interferon-induced protein-10; MCP-1, monocyte chemotactic protein-1; VEGF, vascular endothelial growth factor*.

Non-significant correlations were identified between any factors' levels in the aqueous humor and the CRT value in the vitrectomized group (Table 3). In the non-vitrectomized group, the aqueous humor levels of VEGF were significantly correlated with the CRT values, whereas IL-6, IL-8, IP-10, and MCP-1 in the aqueous humor were not significantly correlated with the CRT values (Table 4).

**Table 3:** Correlations between factor levels in the aqueous humor and CRT in vitrectomized group.

	**ρ**	***p***
IL-6	0.352	0.035
IL-8	0.068	0.695
IP-10	0.248	0.145
MCP-1	0.171	0.319
VEGF	−0.004	0.983

*ρ = correlation coefficient*.

*P = probability value*.

*CRT, central retinal thickness; IL-6, interleukin-6; IL-8, interleukin-8; IP-10, interferon-induced protein-10; MCP-1, monocyte chemotactic protein-1; VEGF, vascular endothelial growth factor*.

**Table 4:** Correlations between factor levels in the aqueous humor and CRT in the non-vitrectomized group.

	**ρ**	***p***
IL-6	0.231	0.053
IL-8	0.216	0.070
IP-10	0.194	0.105
MCP-1	0.147	0.222
VEGF	0.333	0.005^*^

*ρ = correlation coefficient*.

*p = probability value*.

* *Statistical significance: p < 0.007 after Ryan–Holm step-down Bonferroni correction*.

*CRT, central retinal thickness; IL-6, interleukin-6; IL-8, interleukin-8; IP-10, interferon-induced protein-10; MCP-1, monocyte chemotactic protein-1; VEGF, vascular endothelial growth factor*.

In the vitrectomized group, the aqueous humor levels of IL-6 were significantly correlated with the aqueous humor levels of IL-8, IP-10, and MCP-1. The levels of IL-8 in the aqueous humor were significantly correlated with the levels of IP-10 and MCP-1 in the aqueous humor. However, the levels of VEGF were not significantly correlated with any of the measured inflammatory factors (Table 5). In the non-vitrectomized group, the aqueous humor levels of IL-6 were significantly correlated with the aqueous humor levels of IL-8, IP-10, MCP-1, and VEGF. The levels of IL-8 in the aqueous humor were significantly correlated with the levels of IP-10, MCP-1, and VEGF in the aqueous humor. The aqueous humor levels of IP-10 were significantly correlated with the aqueous humor levels of MCP-1 (Table 6).

**Table 5:** Correlation matrix for aqueous humor factors in the vitrectomized group.

**Variable**	**IL-6**	**IL-8**	**IP-10**	**MCP-1**	**VEGF**
	**ρ**	**ρ**	**ρ**	**ρ**	**ρ**
	***p***	***p***	***p***	***p***	***p***
IL-6	–	0.662	0.537	0.775	0.155
		<0.001^*^	0.001^*^	<0.001^*^	0.365
IL-8	–	–	0.564	0.729	0.075
			<0.001^*^	<0.001^*^	0.665
IP-10	–	–	–	0.551	−0.020
				<0.001^*^	0.909
MCP-1	–	–	–	–	0.104
					0.545

*ρ = correlation coefficient*.

*p = probability value*.

* *Statistical significance: p < 0.005 after Ryan–Holm step-down Bonferroni correction*.

*IL-6, interleukin-6; IL-8, interleukin-8; IP-10, interferon-induced protein-10; MCP-1, monocyte chemotactic protein-1; VEGF, vascular endothelial growth factor*.

**Table 6:** Correlation matrix for aqueous humor factors in the non-vitrectomized group.

**Variable**	**IL-6**	**IL-8**	**IP-10**	**MCP-1**	**VEGF**
	**ρ**	**ρ**	**ρ**	**ρ**	**ρ**
	***p***	***p***	***p***	***p***	***p***
IL-6	–	0.647	0.581	0.718	0.479
		<0.001^*^	<0.001^*^	<0.001^*^	<0.001^*^
IL-8	–	–	0.522	0.676	0.583
			<0.001^*^	<0.001^*^	<0.001^*^
IP-10	–	–	–	0.631	0.233
				<0.001^*^	0.05
MCP-1	–	–	–	–	0.238
					0.046

*ρ = correlation coefficient*.

*P = probability value*.

* *Statistical significance: p < 0.007 after Ryan–Holm step-down Bonferroni correction*.

*IL-6, interleukin-6; IL-8, interleukin-8; IP-10, interferon-induced protein-10; MCP-1, monocyte chemotactic protein-1; VEGF, vascular endothelial growth factor*.

The mean interval between vitrectomy and the anti-VEGF intravitreal injection in the vitrectomized group was 17.8 weeks (range 4–78 weeks), and none of the inflammatory or VEGF levels were associated with the days after PPV (*p* > 0.05).

## Discussion

It was the first study to investigate the inflammatory and angiogenic factors in vitrectomized DME eyes. The duration of diabetes in the vitrectomized group was longer than that in the non-vitrectomized group. A longer duration of diabetes might induce severe PDR, resulting in the vitrectomized patients receiving PPV prior to this study. Still, the HbA1c levels were lower in the vitrectomized group than in the non-vitrectomized group, which might be due to the strict control of blood glucose among patients who underwent surgery.

The ocular microenvironment is likely to change after PPV, resulting in a different effect for anti-VEGF treatment in vitrectomized DME eyes. We found that the inflammatory factors including IL-6, IL-8, IP-10, and MCP-1 levels were higher in vitrectomized DME eyes than in non-vitrectomized DME eyes. In contrast, the levels of VEGF were lower in vitrectomized DME eyes than in non-vitrectomized DME eyes.

VEGF is vital to vascular leakage, leading to macular edema (16). In patients with DME, the aqueous humor levels of VEGF are associated with the severity of macular edema (17). In treatment-naïve DME eyes, the levels of VEGF in the aqueous humor were high and were significantly correlated with the CRT, consistent with previous studies (12, 17). The levels of VEGF were lower in vitrectomized DME eyes than in non-vitrectomized DME eyes, which might be due to the removal of VEGF by the PPV procedure. Yanyali et al. (3) showed that anti-VEGF therapy did not affect visual acuity or foveal thickness in vitrectomized eyes with DME. This phenomenon might be due to the reduced contribution of VEGF to DME in vitrectomized eyes. Inflammation might play a more important role in vitrectomized DME eyes than VEGF.

Various cytokines, especially inflammatory factors, form a network that might influence the development and exacerbation of macular edema. We found that the inflammatory factors including IL-6, IL-8, IP-10, and MCP-1 levels were higher in vitrectomized DME eyes than in non-vitrectomized DME eyes.

In this study, the levels of IL-6 in the aqueous humor were higher in the vitrectomized group than in the non-vitrectomized group. This indicates that IL-6 is probably important to the pathogenesis of DME. Indeed, elevated levels of IL-6 in the aqueous humor were associated with macular fluorescein leakage (6), and the recurrence of DME after anti-VEGF treatment has been associated with elevated aqueous humor levels of IL-6 but not with VEGF levels (7).

Our results showed that the levels of IL-8 in the aqueous humor were significantly higher in the vitrectomized group than in the non-vitrectomized group, indicating that DME in vitrectomized eyes might also be associated with IL-8. Previous studies showed that IL-8 enhances inflammation and stimulates angiogenesis by binding its receptor and inducing downstream signaling. Some studies have demonstrated that the levels of IL-8 in the aqueous humor are elevated in DME patients compared with patients without DM (7, 9), supporting a role of IL-8 in the development of DME and supporting the results of the present study.

In this study, the IP-10 levels in the aqueous humor were significantly higher in the vitrectomized group than in the non-vitrectomized group, which might be due to the relationship with IL-8 and the reaction to PRP. Indeed, the levels of IP-10 in the aqueous humor have been significantly correlated with the levels of IL-8 (12), and IP-10 can prevent IL-8-induced neovascularization in a corneal pocket model of angiogenesis (11). These studies indicated that IP-10 might at least partially antagonize the function of IL-8. The levels of IP-10 in the aqueous humor were significantly higher in PDR patients who underwent PRP than who did not receive PRP, indicating that IP-10 might play a role in the inhibition of PDR progression (12).

Our results suggested that the levels of MCP-1 were significantly higher in the vitrectomized group than in the non-vitrectomized group, indicating that MCP-1 might be an important modulator after PPV, especially in DME eyes. MCP-1 levels in the aqueous humor were elevated in DME patients (15), and mean vitreous levels of MCP-1 were significantly higher in vitrectomized eyes with DME than in eyes without DME (18), supporting the present study. Even though clinical examinations showed no inflammatory responses in the eye, MCP-1 levels were still elevated (19).

The high concentrations of inflammatory factors in vitrectomized eyes may be related to the disruptions of the blood–aqueous barrier by surgery (20). Still, a study showed no differences in IL-6, IL-8, MCP-1, and VEGF levels in aqueous humor between PDR patients with and without PRP (12). Another study also found that some inflammatory factors (IL-8 and IP-10) were increased in the aqueous humor of post-vitrectomy eyes in rhegmatogenous retinal detachment patients, whereas other factors (IL-6 and MCP-1) remained unchanged (21). This phenomenon might indicate that some inflammatory factors originate from intraocular cells or tissues. In addition, several studies have found that IL-6, IL-8, IP-10, and MCP-1 are associated with DME; therefore, the elevation of these inflammatory factors might not simply be due to intraocular surgery but also be the result of DME pathology in vitrectomized eyes.

In non-vitrectomized DME eyes, the levels of IL-6, IL-8, and VEGF were significantly correlated with each other, likely because these cytokines are essential to DME pathogenesis and interact among themselves. In vitrectomized DME eyes, IL-6, IL-8, IP-10, and MCP-1 levels were significantly associated; however, VEGF levels in the aqueous humor were not correlated with any inflammatory factors. These findings indicated that inflammation might influence macular edema along with VEGF in non-vitrectomized DME eyes, whereas inflammation might play a more important role in the pathogenesis of DME in vitrectomized eyes *via* the VEGF-independent pathway. Thus, anti-VEGF treatment might not represent an effective treatment for some vitrectomized DME eyes (3).

The evidence shows that DR is mediated by inflammatory responses, including leukostasis (22). The inflammatory cascade appears to be crucial for the occurrence and development of DME. The treatment of DME using intravitreal injections of corticosteroids has been reported to be safe and effective (23). Corticosteroids inhibit the synthesis of multiple inflammatory proteins that might be responsible for the development of vascular leakage (24). The effects of corticosteroid injections for DME in vitrectomized eyes are worth considering.

In this study, non-vitrectomized eyes received intravitreal injections before PRP, while vitrectomized eyes received PRP during PPV. Oh et al. (12) showed that in PDR patients the levels of IL-6, IL-8, MCP-1, and VEGF had no significant differences between eyes that underwent PRP or not, except for IP-10.

The mean interval between PPV and intravitreal injection (17.8 weeks) did not have a significant relationship with any of the cytokines measured in this study. Therefore, the interval after vitrectomy in this study did not influence the results.

One limitation of our study is that we did not examine all cytokines, and the ultimate effects of these cytokines in vitrectomized DME eyes remain to be explored. Moreover, the number of samples in our study was limited.

## Conclusion

In conclusion, this study showed that the levels of inflammatory factors in the aqueous humor were higher, whereas the VEGF levels were lower in vitrectomized DME eyes than in non-vitrectomized DME eyes. In addition, inflammatory factor levels had no relationship with VEGF levels in vitrectomized DME eyes. These findings indicate that inflammation might play an important role in the pathogenesis of DME in vitrectomized eyes. Anti-inflammatory therapies might represent another strategy for the treatment of DME in vitrectomized eyes. Further studies are needed to investigate the mechanism and treatment strategy of DME in vitrectomized eyes.

## Data Availability Statement

The raw data supporting the conclusions of this article will be made available by the authors, without undue reservation.

## Ethics Statement

Our study was approved by the Ethical Committee of The Second Xiangya Hospital (LYZ2020009), and all enrolled patients were treated in accordance with the Declaration of Helsinki. All patients provided informed consent before inclusion in the study. To reduce selection bias, all patients were recruited in a consecutive manner.

## Author Contributions

YL was responsible for designing the study, conducting the search, screening potentially eligible studies, extracting and analyzing the data, interpreting the results, and writing the paper. BY was responsible for extracting data. ZM, MX, ZL, ZZ, YM, JM, and BM were responsible for extracting data. XY was responsible for the data collection and modifying the language. JL was responsible for designing the study. All authors contributed to the article and approved the submitted version.

## Funding

This work was supported by the National Natural Science Foundation of China (81570847), Natural Science Foundation of Hunan Province (2020JJ4800).

## Conflict of Interest

The authors declare that the research was conducted in the absence of any commercial or financial relationships that could be construed as a potential conflict of interest.

## Publisher's Note

All claims expressed in this article are solely those of the authors and do not necessarily represent those of their affiliated organizations, or those of the publisher, the editors and the reviewers. Any product that may be evaluated in this article, or claim that may be made by its manufacturer, is not guaranteed or endorsed by the publisher.
